# Investigation of LiF, Mg and Ti (TLD-100) Reproducibility

**Published:** 2015-12-01

**Authors:** M. Sadeghi, S. Sina, R. Faghihi

**Affiliations:** 1 Nuclear Engineering Department, School of Mechanical Engineering, Shiraz University, Shiraz, Iran; 2 Radiation Research Center, School of Mechanical Engineering, Shiraz University, Shiraz, Iran

**Keywords:** LiF, Mg, and Ti, TLD-100, ECC, Reproducibility

## Abstract

LiF, Mg and Ti cubical TLD chips (known as TLD-100) are widely used for dosimetry purposes. The repeatability of TL dosimetry is investigated by exposing them to doses of (81, 162 and 40.5 mGy) with 662keV photons of Cs-137. A group of 40 cubical TLD chips was randomly selected from a batch and the values of Element Correction Coefficient (ECC) were obtained 4 times by irradiating them to doses of 81 mGy (two times), 162mGy and 40.5mGy. Results of this study indicate that the average reproducibility of ECC calculation for 40 TLDs is 1.5%, while these values for all chips do not exceed 5%.

## Introduction

LiF, Mg and Ti thermoluminescent dosimeters (known as TLD-100) are common dosimeters in different fields of dosimetry like diagnostic radiology[1-3], radiation therapy[4-7] and personal monitoring[8]. A good knowledge of optimum dosimetry procedures is necessary for performing exact dosimetry. Energy, dose rate and angular dependency pre- and post- irradiation fading, annealing, optimum time temperature profile for TLD readout and radiation field homogeneity are very important issues in optimization of TLD responses[9-14] The aim of this study is to investigate the reproducibility in the response of TLD 100 cubical chips.

## Material And Methods

### LiF, Mg, Ti (TLD 100)

Lithium Fluoride LiF is an alkali halide widely used in constructing personal dosimeters like LiF, Mg, Ti (TLD-100) and LiF, Mg, Cu and P (TLD-100H). TL dosimeters are used in forms of powders, cubical or cylindrical chips, rods, etc. TLD100 chips are LiF crystals doped with titanium and magnesium to increase the number of traps and luminescence centers. TLD100 chips are common dosimeters in medical and environmental dosimetry. Cubical TLD chips with dimensions of 3.1mm×3.1mm×1mm were used in this investigation for reproducibility studies.

### Reproducibility of TL Response

40 TLD100 chips were randomly selected from a TLD batch and were annealed with a standard annealing procedure (first heated at 400°C for an hour and then at 80°C for 20 hours). All TLD were exposed to equal dose of 81mGy by 662 keV photons of Cs-137 source (i.e. an hour irradiation at dose rate of 1.35 mGy/min). TLD chips were read out using Harshaw 4500 TLD reader one day after irradiation. The time-temperature profile used in this study for TLD read out is shown in Table 1.

**Table 1:** Time-temperature profile (TTP) used for TLD100 read out

**Dosimeter**	**TTP**	
LiF: Mg, Ti TLD-100	**Preheat**	
	Temp (◦C)	50
	Time (sec)	0
	**Acquisition**	
	Max Temp (◦C)	300
	Time (sec)	13.33
	Rate (◦C/sec)	25
	**Anneal**	
	Temp (◦C)	0
	Time (sec)	0

To test the reproducibility of Thermoluminescence dosimetry, the above mentioned procedure was repeated 4 times. According to Moor et al (2008), the Coefficient of Variation (CV) of TLD measurement which is obtained by equation 1, should not exceed 10%.

$$CV\left(\%\right)=\frac{SD}{mean}\times 100\;(1)$$

### Reproducibility in ECC Calculations

Element Correction Coefficient (ECC) of a TLD is a correction factor that relates the thermoluminescence efficiency (TLE) of each dosimeter to the average TLE of all dosimeters (<TLE>) exposed to equal dose, as follows:

$${ECC}_{i}=\frac{<TLE>}{{TLE}_{i}}\;(2)$$

Where *ECC_i_* is the *ECC* of a dosimeter *i*, <*TLE*> is the mean *TLE* of the dosimeters, and *TLE_i_* is the *TLE* of dosimeter *i*.

A group of 40 TLDs was exposed to 81mGy of Cs-137 gamma rays (1 hour exposure to the dose rate of 1.35mGy/min). ECC values of all chips were obtained according to equation 2.

To check the reproducibility of ECC calculation of TLD-100 dosimeters, ECC values were obtained once again by exposing TLDs to 81 mGy dose. To investigate the effect of dose on the element calibration coefficient, ECC values were obtained by exposing TLDs to 162 and 40.5mGy, (0.5 hour and 2 hours exposure to dose rate of 1.35 mGy/min, respectively). Finally, four ECC values obtained for TLDs were compared for each TL dosimeter and the average ECC and standard deviation of the mean were obtained. 

## Results and Discussions

### Reproducibility of TL Response

**Figure 1:**
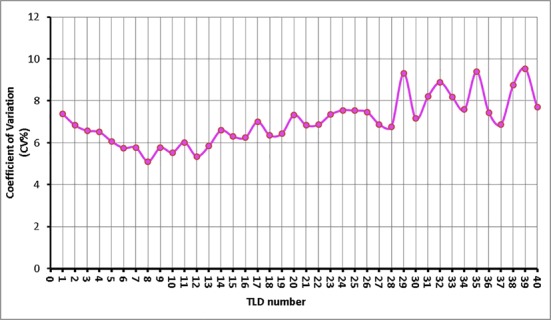
The coefficient of variation (CV%) for the response of each TLD

The coefficient of variation (CV) for the response of each chip was obtained for four measurements. To calculate CV, mean value and standard deviation of the four readings were calculated for each TLD. Figure 1 shows the (CV%) for each TLD. As it is obvious from the figure, values of CV for all chips are less than 10%.

To omit the effect of systematic error in TLD responses, the reproducibility of element correction coefficient, which is a relative dose, was checked.

### Reproducibility in ECC Calculations

**Figure 2:**
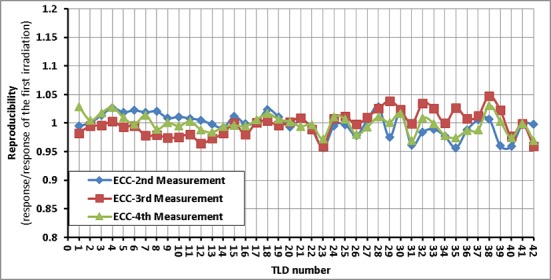
Reproducibility of the ECC

**Figure 3:**
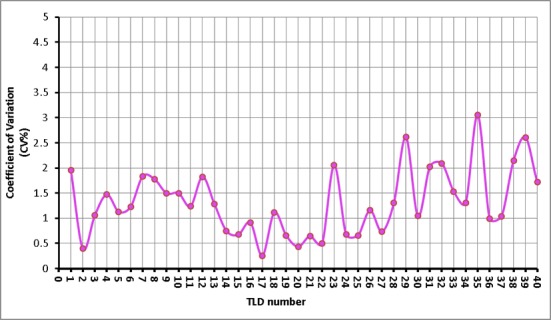
Coefficient of variation (CV%) for the ECC of each TLD

Table 2 shows the percentage difference between ECC values in each measurement and the ECC values in the first measurement. This table indicates that the percentage differences between ECC values are less than 5% for all TLDs. The average value and the standard deviation of the ECC obtained by four measurements were calculated for each TLD. According to these results, the standard deviations of the mean values for ECC vary between 0.003 and 0.041. Figures 2 and 3 show the reproducibility of ECC calculations and the coefficient of variation (CV%). Comparing the reproducibility of raw responses with the reproducibility of ECC values shows that the repeatability in ECC values is better than the repeatability in TLD responses. This is because of the fact that ECC values are relative quantities and systematic error due to other sources of errors (exposure time, reader stability, TLD positioning, etc) are deleted. According to figures1 and 3, the coefficient of variation (CV%) is found to be less than 9.53%  and 3.06% for TL response and ECC calculations, respectively, which are less than 10% for all TLDs.

**Table 2:** Percentage difference between ECC values

	**% difference between 1st and 2nd measurement**	**% difference between 1st and 3rd measurement**	**% difference between 1st and 4th measurement**
minimum	0.05	0.02	0.05
maximum	4.38	4.75	3.09
average	1.63	1.65	1.17

## Conclusion

The repeatability of measurements with Thermoluminescence dosimeters is one of the most important issues which should be taken into consideration. The reproducibility of response of 40 TLD chips which had been previously used for several measurements, was checked in this investigation. Results of this study indicate that the reproducibility in thermoluminescence dosimetry should be improved by reduction of error sources as much as possible. For instance, the systematic error observed in TLD responses may be due to the errors in positioning of TLDs for irradiation. 
